# Anti-VEGF therapy prevents Müller intracellular edema by decreasing VEGF-A in diabetic retinopathy

**DOI:** 10.1186/s40662-021-00237-3

**Published:** 2021-04-17

**Authors:** Tianqin Wang, Chaoyang Zhang, Hai Xie, Mengmeng Jiang, Haibin Tian, Lixia Lu, Guo-Tong Xu, Lin Liu, Jingfa Zhang

**Affiliations:** 1 Department of Ophthalmology, Renji Hospital, Shanghai Jiao Tong University School of Medicine, 160 Pujian Road, Pudong New District, Shanghai, 200127 China; 2 Department of Ophthalmology, Shanghai General Hospital (Shanghai First People’s Hospital), Shanghai Jiao Tong University, 100 Haining Road, Hongkou District, Shanghai, 200080 China; 3 National Clinical Research Center for Eye Diseases; Shanghai Key Laboratory of Ocular Fundus Diseases, Shanghai Engineering Center for Visual Science and Photomedicine, Shanghai Engineering Center for Precise Diagnosis and Treatment of Eye Diseases, Shanghai, China; 4 Tongji Eye Institute, Tongji University School of Medicine, 1239 Siping Road, Medical School Building, Room 623, Shanghai, 200092 China

**Keywords:** Diabetic retinopathy, Diabetic macular edema, Müller cell, Anti-VEGF, Intracellular edema

## Abstract

**Background:**

Although vascular endothelial growth factor A (VEGF-A) is known to play a key role in causing retinal edema, whether and how VEGF-A induces intracellular edema in the retina still remains unclear.

**Methods:**

Sprague-Dawley rats were rendered diabetic with intraperitoneal injection of streptozotocin. Intravitreal injection of ranibizumab was performed 8 weeks after diabetes onset. rMC-1 cells (rat Müller cell line) were treated with glyoxal for 24 h with or without ranibizumab. The expression levels of inwardly rectifying K^+^ channel 4.1 (Kir4.1), aquaporin 4 (AQP4), Dystrophin 71 (Dp71), VEGF-A, glutamine synthetase (GS) and sodium-potassium-ATPase (Na^+^-K^+^-ATPase) were examined using Western blot. VEGF-A in the supernatant of the cell culture was detected with ELISA. The intracellular potassium and sodium levels were detected with specific indicators.

**Results:**

Compared with normal control, protein expressions of Kir4.1 and AQP4 were down-regulated significantly in diabetic rat retinas, which were prevented by ranibizumab. The above changes were recapitulated in vitro. Similarly, the intracellular potassium level in glyoxal-treated rMC-1 cells was increased, while the intracellular sodium level and Na^+^-K^+^-ATPase protein level remained unchanged, compared with control. However, ranibizumab treatment decreased intracellular sodium, but not potassium.

**Conclusion:**

Ranibizumab protected Müller cells from diabetic intracellular edema through the up-regulation of Kir4.1 and AQP4 by directly binding VEGF-A. It also caused a reduction in intracellular osmotic pressure.

## Background

Diabetic retinopathy (DR) is the leading cause of blindness in working-age people, in which diabetic macular edema (DME) is the most common complication of DR.^1, 2^ Retinal edema, driven by Starling equation, results from the imbalance between fluid entry, fluid exit and retinal hydraulic conductivity, which leads to intraretinal or subretinal fluid accumulation. Under physiological condition, influx and efflux of ions and water are regulated by the integrity of the blood-retinal barrier (BRB) and the normal functioning of Müller cells and the retinal pigment epithelium (RPE).^3^ However, in DME, the increased fluid entry and decreased drainage function result in intracellular and extracellular edema. The breakdown of the inner BRB plays the most important role, caused by junctional complex alternation,^4^ enhanced transcellular permeability,^5^ loss of endothelial cells,^6^ loss of pericytes^7^ and vessel abnormality.^8, 9^ Dysfunction of Müller glia and RPE also contribute to the fluid accumulation in the neural retina and subretinal space leading to intracellular and extracellular edema.^10^ In our previous study, a strong correlation was found between central subfield thickness (CSFT) and the thickness of inner nuclear layer (INLT) in more severe DME (CSFT > 275 μm), suggesting that intracellular edema, particularly Müller glial edema, contributes to DME formation.^11^ Müller cells, as specific macroglia in the retina, regulate the homeostasis of ion and water mainly through inward rectifying potassium channel 4.1 (Kir4.1) and aquaporin 4 (AQP4).^12–14^ Müller cells are also the main source of vascular endothelial growth factor (VEGF) apart from vascular endothelial cells.^15^

The polarized distribution of Kir4.1 enables the efflux of potassium away from the neural retina.^13^ Water, accompanied with potassium and powered by osmotic pressure, is transported through AQP4, a selective water transport protein co-localized with Kir4.1. Both Kir4.1 and AQP4 are anchored by Dystrophin 71 (Dp71) on the membranes of Müller cells.^16, 17^ It was reported that the swelling of Müller cells was caused by the downregulation or redistribution of Kir4.1, AQP4 and Dp71 in many disease models, such as retinal vein occlusion and ischemia-reperfusion injury.^18, 19^

**Fig. 1:**
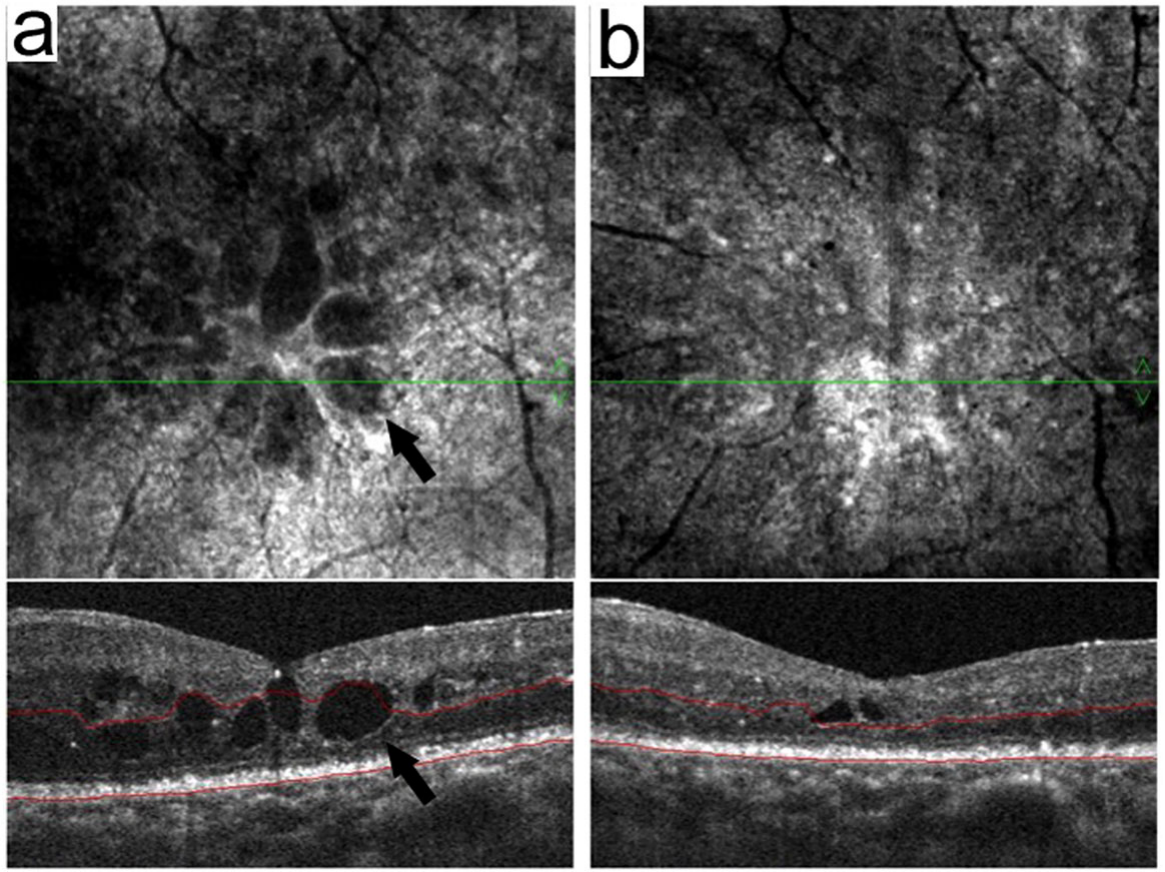
Diabetic macular edema (DME) examined with OCT-A before and after anti-VEGF therapy. Images of one patient with DME (**a**) before and (**b**) after treatment were shown. The arrows on both en-face and b-scan views show Müller intracellular edema (**a**), which was decreased significantly 1 week after anti-VEGF treatment (**b**)

Since VEGF acts as a key mediator in DME pathogenesis, intravitreal anti-VEGF therapy has become the standard of care in treating DME. In patients with DR, cystoid DME is believed to be formed by swollen and dying Müller cells.^20^ In our clinical practice, optical coherence tomography angiography (OCT-A) showed cystoid edema on both en-face and b-scan views in DME patients (Fig. 1a), highlighting Müller intracellular edema.^20^ One week after intravitreal anti-VEGF injection, the cystoid edema was decreased dramatically with the reduced number and size of cystoids (Fig. 1b), implying a potential effect of anti-VEGF therapy on the reduction of Müller intracellular edema apart from its classical effect on non-cellular edema reduction. However, the possible mechanism(s) for anti-VEGF effect still remains unknown, e.g. whether or not anti-VEGF treatment could directly affect the expression levels or distribution of Kir4.1, AQP4 and Dp71 in Müller cells in the diabetic retina. Therefore, in this study, we aimed to explore the possible mechanism(s) of ranibizumab in protecting Müller cells from intracellular edema in experimental DR.

## Material and methods

### Reagents and antibodies

The primary antibodies against Kir4.1 (APC-035) and AQP4 (AQP-004) were from Alomone Lab (Jerusalem, Israel). Dystrophin (ab7164), GFAP (ab53554) and Na^+^-K^+^-ATPase (ab76020) antibodies were from Abcam (Cambridge, UK). Glutamine synthetase (GS, NBP2–43646) and VEGF-A (NB100–664) antibodies were from Novus Biological (Littleton, USA). Potassium indicator (P1267MP) and sodium indicator (S1263) were from Thermo Fisher Scientific (Shanghai, China).

### Experimental animals and intravitreal ranibizumab injection

The animals were treated in compliance with the ARVO Statement for the Use of Animals in Ophthalmic and Vision Research and the Guides for the Care and Use of Animals (National Research Council and Tongji University). The protocol was approved by the ethics committee of Animal Experiments of Tongji University (Permit Number: TJHBLAC-2020-06). Sixty male Sprague-Dawley rats weighing 120g to 150 g (Slaccas, Shanghai, China) were divided into three groups: normal control (N), diabetic rats (D), and diabetic rats treated with intravitreal ranibizumab (D + R). Diabetes was induced by intraperitoneal injection of STZ (60 mg/kg body weight, dissolved in citrate buffer, pH 4.5) and the control rats received an equal volume of citrate buffer. Rats with blood glucose level exceeding 300 mg/dL for 3 consecutive days were considered as diabetic rats and included in this study. Intravitreal injection was performed in diabetic rats 8 weeks after diabetes onset. Ranibizumab (20 μg/eye in a 2 μL) was injected intravitreally with a microsyringe (Hamilton, Reno, NV, USA) through a 30-gauge, 0.5-in needle (BD Biosciences, Franklin Lakes, NJ, USA). For normal and diabetic controls, the same volume (2 μL) of normal saline was injected. Four weeks after the injection, the rats were sacrificed and the eyes were enucleated for the following study.

### Rat Müller cell (rMC-1) culture

Transformed rat retinal Müller cell line (rMC-1) was kindly supplied by Sarthy (Northwestern University, Chicago, IL, USA). The cells were cultured in low glucose (5.5 mM) DMEM containing 10% fetal bovine serum (Gibco, Shanghai, China) and 1% penicillin/streptomycin at 37 °C with 5% CO_2_ in a humidified incubator. The cells were divided into three groups, i.e., normal control (N), glyoxal (1 mM)-treated group (G), and glyoxal (1 mM) + ranibizumab (0.125 mg/mL)-treated group (G + R).

### Cell viability assay

Cell viability of rMC-1 cells was measured using the Cell Counting Kit-8 (CCK-8) assay. Briefly, the rMC-1 cells, incubated with different doses of glyoxal (0.1 to 5 mM), were seeded on 96-well plates at a density of 10^4^ cells per well treated with or without ranibizumab (0.125 mg/mL) for 1h to 36 h, then incubated with DMEM containing 10% of CCK-8 for 3 h at 37 °C. The absorbance was measured at 450 nm by using a microplate spectrophotometer (Tecan, Crailsheim, Germany). The cell viability was expressed as the percentage of the untreated control, which was defined as 100% for each experiment.

### RNA extraction and real-time PCR

Total RNA was extracted from rMC-1 cells. Reverse transcription was performed and real-time PCR was carried out using SYBR Green Real-Time PCR master mix (Toybo, Osaka, Japan). The primer information was listed in Table 1.

**Table 1:** Primer information

Primer name		Sequence	Product size (bp)
Kir4.1	Sense	5′ -TTACAGCCAGACGACGCAGACA- 3’	243
	Antisense	5′ -ACCAGATACCACACCACGCCAA- 3’	
AQP4	Sense	5′ -GGAAGGCATGAGTGACGGAG- 3’	248
	Antisense	5′ -TGCTGAGTCCAAAGCAGAGG- 3’	
Dp71	Sense	5′ -ATGAGGGAACAGCTCAAAGG- 3’	183
	Antisense	5′ -TGCAGCTGACAGGCTCAAGA- 3’	
GS	Sense	5′ -CAGAGACCAACCTGAGGCACAG- 3’	175
	Antisense	5′ -GCTCCCACACCGCAGTAATAGG- 3’	
VEGF-A	Sense	5′ -GCACATAGGAGAGATGAGCTTCC- 3’	105
	Antisense	5′ -CTCCGCTCTGAACAAGGCT- 3’	
β-Actin	Sense	5′ -GTAAAGACCTCTATGCCAACA- 3’	227
	Antisense	5′ -GGACTCATCGTACTCCTGCT- 3’	

### Western blot

Equal amounts of protein were resolved on 10% SDS-polyacrylamide gels and transferred electrophoretically onto nitrocellulose membranes (Bio-Rad, Shanghai, China). The membranes were blocked in 5% Tris buffered saline Tween-20 (TBST) buffered bovine serum albumin at room temperature for 30 min, and then incubated separately with antibodies against Kir4.1 (1: 500), AQP4 (1: 1000), Dystrophin 71 (1: 1000), GS (1: 1000), GFAP (1: 1000), VEGF-A (1: 1000), Na^+^-K^+^-ATPase (1: 10,000) or β-actin (1: 2000), overnight at 4 °C and with the corresponding secondary antibodies (1: 10,000) at room temperature for 1 h. The membranes were visualized by chemiluminescence or Odyssey infrared imaging system (LICOR Biosciences, Lincoln, NE, USA). The optical density of each band was determined using Quantity One software (Bio-Rad), and the densitometric values for the proteins were normalized by β-actin.

### Evaluation of Müller intracellular edema with semithin section

Intracellular edema of retinal Müller cell in vivo was evaluated according to the published method.^21^ Briefly, the rats were killed, the eyes were enucleated and fixed in 2.5% glutaraldehyde for 30 min. The eyes were dissected under a dissecting microscope and the anterior parts of the eye were removed. The posterior part was fixed in 2.5% glutaraldehyde for 5 more hours, which were then dehydrated in a graded alcohol series (50, 70, 95, and 100%) and embedded in epoxy resin for sectioning. During the sample preparation of semithin section, the retinas were cut into several small pieces about 2.25 mm^2^. Semithin sections (1 μm) were cut using an ultramicrotome (EM UC7, Leica, Germany) and stained with toluidine blue. The morphology of swollen Müller cell was examined under a light microscope (Tecnai G2 20 TWIN, FEI, USA). For quantitation, the Müller intracellular edema was calculated as the number of ribbon-like gaps per 200 μm of the retina.

### Immunofluorescence

The rat eyes were fixed at 4 °C overnight in 4% PBS-buffered paraformaldehyde. The anterior segments of the eyeball were removed under the dissecting microscope. The remaining eye cups were dehydrated in 30% sucrose solution for 2 days, and then embedded in optimal cutting temperature compound (OCT; Sakura Finetek Japan Co., Ltd., Tokyo, Japan) for section. The sections (15 μm thick) were permeabilized and blocked in PBS buffered 1% BSA and 0.05% Triton X-100 for 1 h. Then the sections were incubated with primary antibodies at 4 °C overnight and with their appropriate secondary antibodies at room temperature for 1 h. After incubation with 4′,6-diamidino-2-phenylindole (DAPI) for 2 min, the slides were mounted with coverslips. Slides were visualized with Leica microscope (DMI3000, Germany). Exposure conditions in the same channel for different groups in each experiment were consistent.

Immunostaining for Kir4.1, GS and GFAP in retinal sections was quantified using image J (http://imagej.nih.gov/ij/; provided in the public domain by the National Institutes of Health, Bethesda, MD) and indicated as the integrated optical density of the fluorescence (OD) per unit length of retina. The defined area for quantification was selected by using rectangle selection tool. The fluorescence signal was adjusted by setting an appropriate threshold which was the same and applied to all the images.

### ELISA

The supernatants of rMC-1 cells were collected and stored at −80 °C until assay. The concentration of VEGF-A in the supernatant was measured with ELISA kits according to the instruction of the manufacture. The VEGF-A concentration, calculated from the standard curve and normalized by the total protein concentration, was expressed as nanogram per microgram of total protein (ng/mg of total protein).

### Measurement of intracellular sodium and potassium levels

Intracellular sodium and potassium concentration were detected with the specific indicator, i.e., SBFI AM (sodium) and PBFI AM (potassium). The stock solution (1 mM) was reconstituted in DMSO, stored in the dark at −20 °C. The rMC-1 cells were first seeded on 96-well plates and treated with glyoxal (1 mM) with or without ranibizumab (0.125 mg/mL) for 24 h. The cells were incubated with SBFI AM or PBFI AM (diluted to the final concentration 10 μM) at 37 °C for 3 h. Fluorescence was measured using a plate reader (excitation = 340 nm, emission = 500 nm). Fluorescence intensity was normalized by the cell number.

### Statistical analysis

The results were expressed as mean ± SE. Statistical analysis was performed with the SPSS software, version 22.0 (IBM Company, Armonk, NY, USA), and one-way ANOVA with Dunnett’s test was used. The *P* value of 0.05 or less was considered statistically significant.

## Results

### Müller intracellular edema was increased in diabetic rat retina, which was alleviated by ranibizumab

**Fig. 2:**
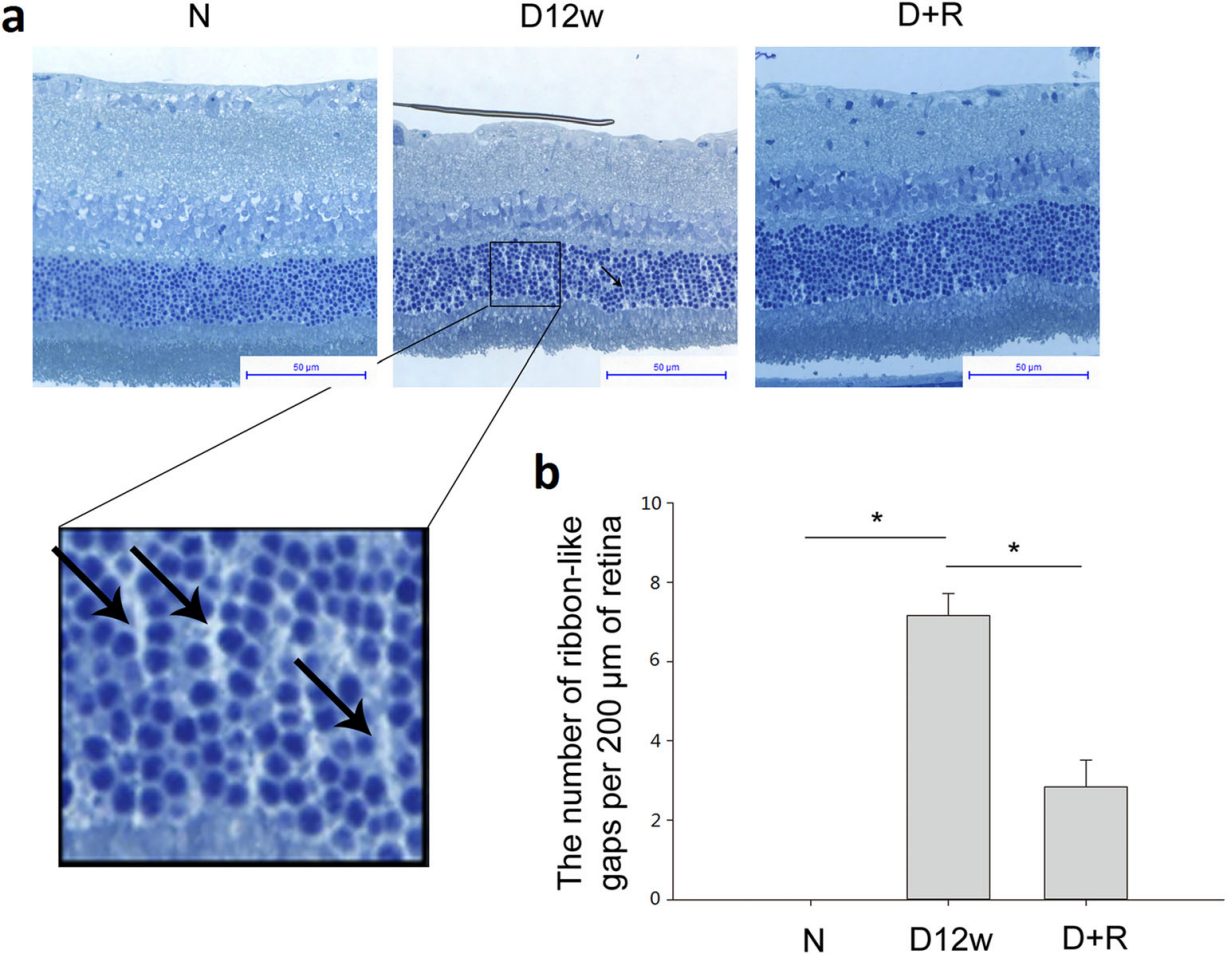
Decrease in fluid accumulation by ranibizumab in the diabetic rat retina. **a** The morphology of normal control (left), diabetic (middle) and ranibizumab treated (right) rat retinas. **b** The ribbon-like transparent gaps, indicated by the arrows, demonstrate the swollen apical processes of Müller cells or their prolongations surrounding extracellular spaces. Müller intracellular edema was calculated as the number of ribbon-like transparent gaps per 200 μm of the retina. n = 6, * *P* < 0.05. N: normal control; D12w: 12-week diabetic retina; D + R: diabetic rat treated with ranibizumab. Scale bar: 50 μm

In order to evaluate Müller intracellular edema in vivo, we adopted the published method by using semithin sections of the retina.^21^ As shown in Fig. 2, compared to normal control group, fluid accumulation was detected between nuclei of the outer nuclear layer (ONL) as ribbon-like transparent gaps, showing the swollen apical processes of Müller cell or their prolongations surrounding extracellular spaces. The intracellular edema of Müller cells was alleviated after ranibizumab treatment (Fig. 2a). As shown in Fig. 2b, no ribbon-like gaps were found in the ONL of the normal control retina, but the number of swollen Müller cells increased significantly (7.17 ± 0.55 per 200 μm, n = 6, *P* < 0.05) in the 12-week diabetic retina, which was largely decreased by 60.5% (2.83 ± 0.68 per 200 μm, n = 6, *P* < 0.05) 4 weeks after Ranibizumab treatment, indicating the potential effect of ranibizumab on the reduction of intracellular edema of Müller cells.

### The expression of Kir4.1 was down-regulated in the rat retina with diabetes progression

**Fig. 3:**
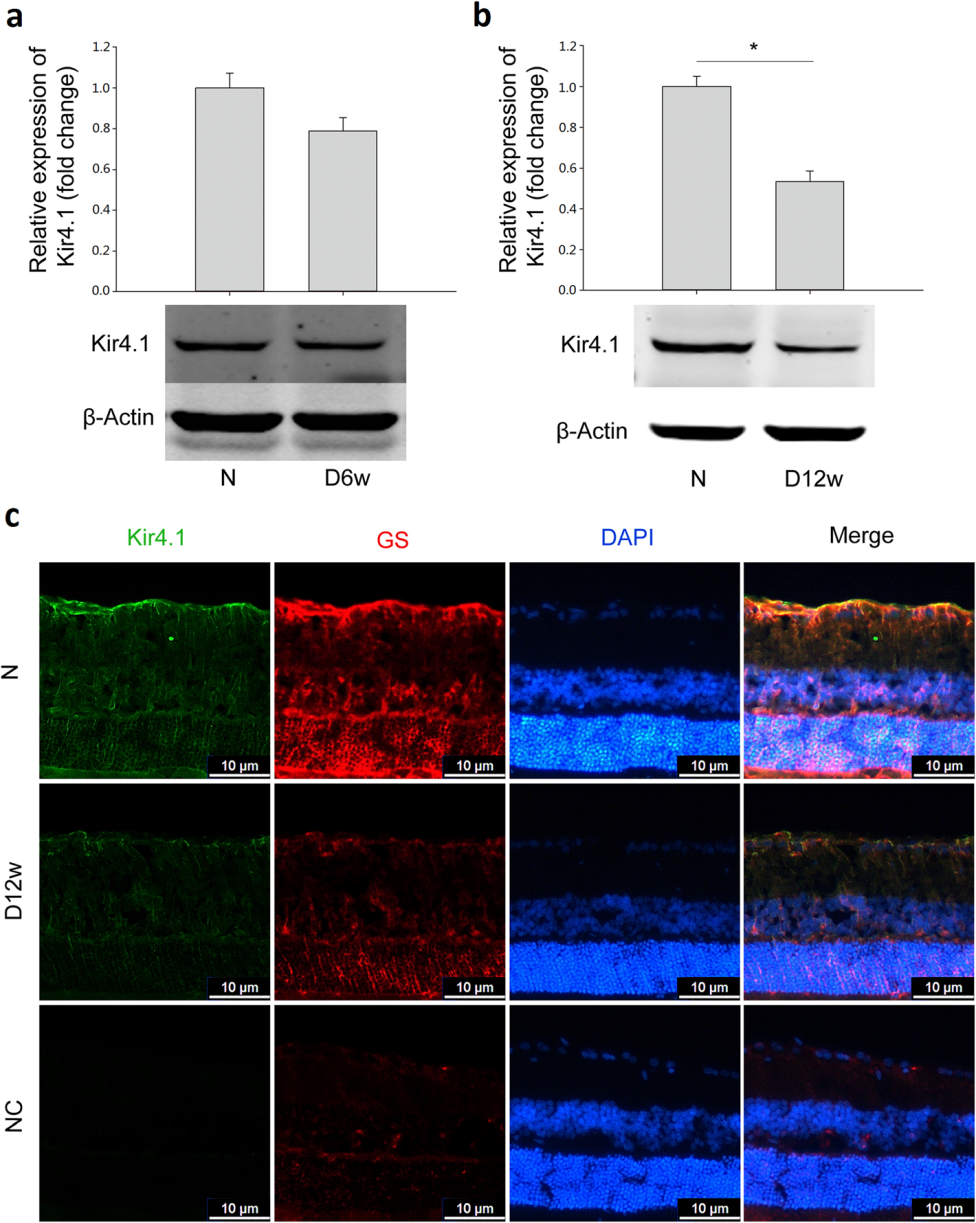
Expression of Kir4.1 in the retina was downregulated with progression of diabetes. The protein expression levels of Kir4.1 in (**a**) 6-week and (**b**) 12-week diabetic rat retinas. **c** Immunostaining of Kir4.1 and GS in 12-week diabetic rat retinas (Kir4.1, green; GS, red; DAPI, blue). Data are expressed as mean ± SE. n = 6 in [a], n = 4 in [b], * *P* < 0.05. N: normal control; D6w: 6-week diabetic retina; D12w: 12-week diabetic retina; NC, negative control. Scale bar: 10 μm

The examination of protein expression of Kir4.1 in diabetic rat retinas showed that, the Kir4.1 level in diabetic rat retinas was decreased by 21.0% at 6 weeks (Fig. 3a) and 46.7% at 12 weeks (Fig. 3b), respectively, when compared with control.

The decreased expression of Kir4.1 in the 12-week diabetic rat retina was also confirmed with immunofluorescence. From Fig. 3c, Kir4.1 in the normal control is mainly expressed in the inner limiting membrane (ILM) and co-localized with GS, a specific marker for Müller cells. However, in diabetic retinas, the distribution of Kir4.1 was largely disrupted, extending from the ILM to the outer limiting membrane (OLM), with weak immunostaining especially in the ILM and around retinal blood vessels. The fluorescence intensities of Kir4.1 and GS were quantified, and compared with that in normal control, the fluorescence intensities were decreased by 57.0% (Kir4.1, n = 5, *P* < 0.05) and 76.0% (GS, n = 3, *P* < 0.05), respectively, in 12-week diabetic rat retinas.

### Ranibizumab increased the expression levels of Kir4.1 and AQP4 in the diabetic rat retina

**Fig. 4:**
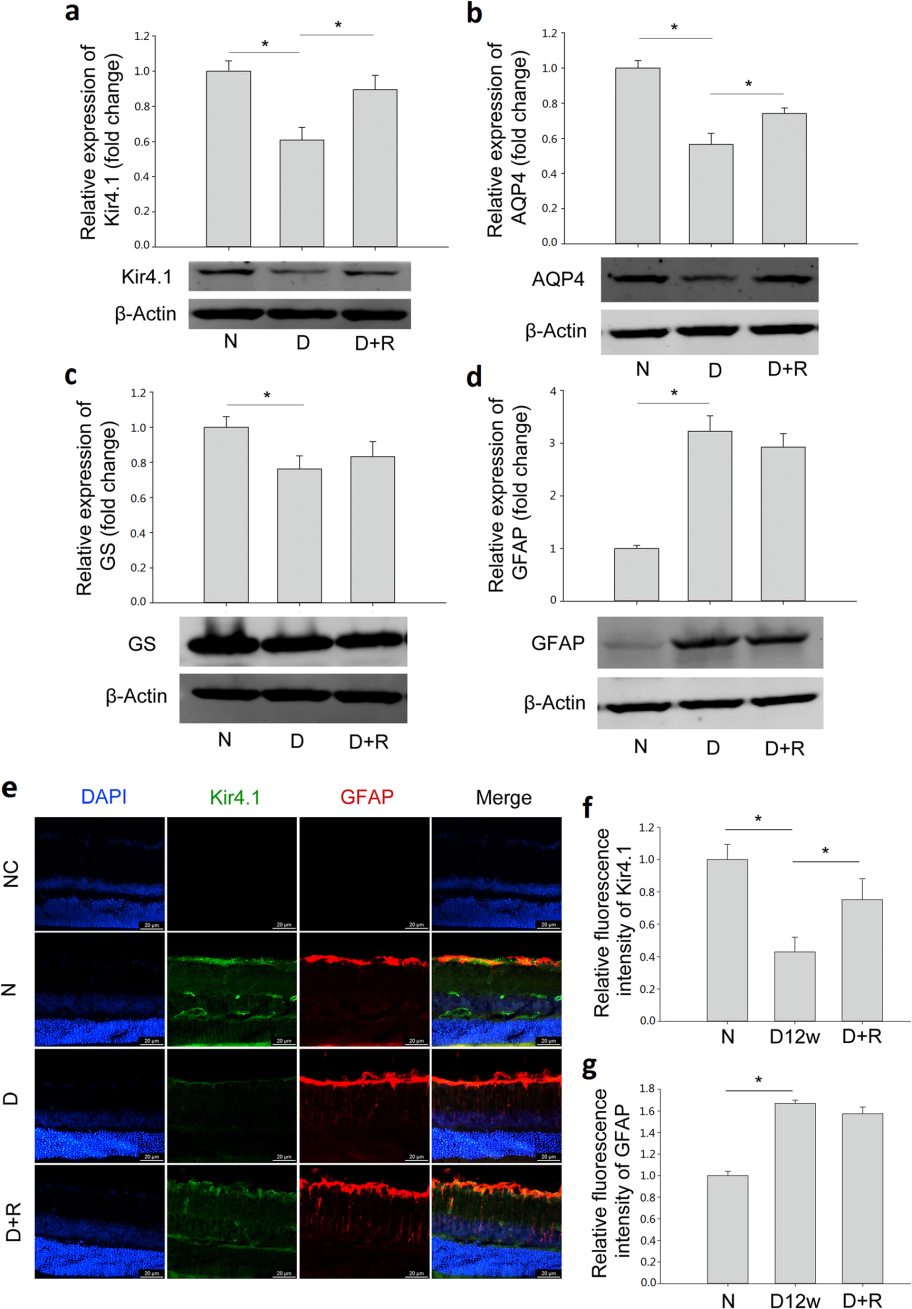
Protein changes of (**a**) Kir4.1, (**b**) AQP4, (**c**) GS, and (**d**) GFAP in diabetic rat retinas treated with or without ranibizumab. **e** Co-immunostaining of Kir4.1 and GFAP in 12-week diabetic rat retinas (Kir4.1, green; GFAP, red; DAPI, blue), scale bar: 20 μm. Data are expressed as mean ± SE (n = 7, *P < 0.05). Quantification of fluorescence intensities of Kir4.1 (f, n = 5, *P < 0.05) and GFAP (g, n = 3, *P < 0.05) among three groups. N: normal control; D: 12-week diabetic retina; D + R: diabetic rat treated with ranibizumab

To test the effect of ranibizumab on the expression levels of Kir4.1 and AQP4, Western blot was performed in 12-week diabetic rat retinas treated with or without ranibizumab. As shown in Fig. 4a, after ranibizumab treatment, the protein level of Kir4.1 was up-regulated by 47.5% compared with that in the diabetic rat without ranibizumab treatment. Similarly, AQP4 expression level in the diabetic group decreased significantly (by 43.3% vs. normal control group), which was up-regulated by 30.9% after ranibizumab treatment (Fig. 4b). The protein expression levels of GS and GFAP in Müller cells of diabetic retinas were also evaluated. GS in diabetic retinas was decreased by 23.7% (Fig. 4c), while GFAP was increased by 222.7% (Fig. 4d), as compared with control, indicating the activation of Müller cells but with decreased ability in metabolizing glutamate. Treatment with ranibizumab had no effect on GS and GFAP expression.

To further confirm the effect of ranibizumab on Kir4.1 and GFAP, we performed double immunostaining of both proteins in diabetic rat retinas treated with or without ranibizumab. As shown in Fig. 4e, in normal control, Kir4.1 was mainly expressed in the ILM and around the vessels, which co-localized with GFAP, another marker of Müller cells. However, in 12-week diabetic rat retinas, the decreased expression of Kir4.1 with altered distribution was observed; there was an attenuated staining pattern especially in the ILM and around vessels. GFAP immunostaining in Müller cells was increased in 12-week diabetic rat retinas with its characteristic radial immunostaining pattern. Ranibizumab treatment increased Kir4.1 expression as well as maintained its distribution, but showed no effect on GFAP (Fig. 4e). The above immunostaining was also quantified, and compared with normal control, the fluorescence intensity of Kir4.1 was decreased by 57.0% (n = 5, *P* < 0.05) in 12-week diabetic rat retinas, which was increased by 75.0% (n = 5, *P* < 0.05) after ranibizumab treatment (Fig. 4f). On the other hand, the fluorescence intensity of GFAP was increased by 67.1% (n = 3, *P* < 0.05) in diabetic retinas, which remained unchanged after ranibizumab treatment (n = 3, *P* > 0.05, Fig. 4g), consistent with the Western blot (Fig. 4d).

### Ranibizumab decreased VEGF-A and increased protein expression levels of Kir4.1, AQP4 and Dp71 in glyoxal-treated rMC-1 cells

**Fig. 5:**
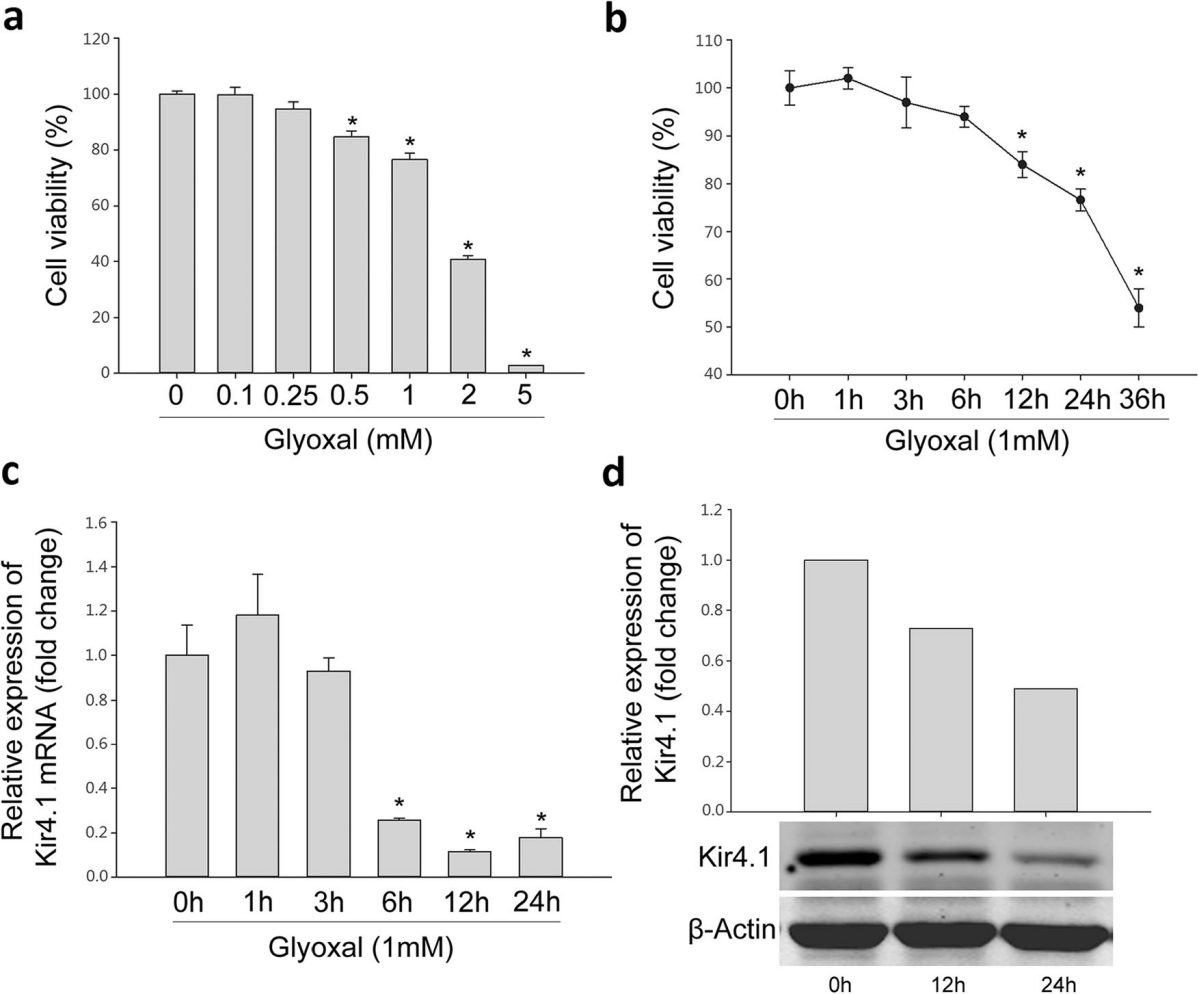
Downregulation of Kir4.1 was induced by glyoxal on rMC-1 cells. **a** Cell viability of rMC-1 cells treated with different doses of glyoxal. **b** Cell viability of 1 mM glyoxal treated-rMC-1 cells at different time points. The (**c**) mRNA and (**d**) protein expression level of Kir4.1 in rMC-1 cells treated with 1 mM glyoxal. Data are expressed as mean ± SE. n = 12 in [a, b], n = 6 in [c], n = 1 in [d], *P < 0.05

To further confirm above observation, we adopted glyoxal-treated rMC-1 cells to mimic the diabetic condition. As shown in Fig. 5, the rMC-1 cells were treated with different doses of glyoxal (Fig. 5a) for different time points (Fig. 5b) to optimize the glyoxal treatment conditions. Cell viability was decreased in a dose-dependent manner by 0.3% (0.1 mM), 5.2% (0.25 mM), 15.3% (0.5 mM), 23.4% (1 mM), 59.2% (2 mM) and 97.4% (5 mM) when treated with different doses of glyoxal for 24 h. When the cells were treated with glyoxal (1 mM) at different time points, cell viability was transiently increased by 2% (1 h) then decreased by 3.1% (3 h), 6.4% (6 h), 16.1% (12 h), 23.4% (24 h) and 46.0% (36 h). Based on our cell viability assay, we chose 1 mM of glyoxal and 24 h treatment for the following study.

When rMC-1 cells were treated with glyoxal (1 mM), the mRNA expression level of Kir4.1 was about 118.1% (1 h), 93% (3 h), 25.6% (6 h), 11.5% (12 h), 17.7% (24 h) compared with normal control (Fig. 5c). Furthermore, Western blot demonstrated that Kir4.1 protein level was decreased by 27.2 and 51.0% at 12 and 24 h, respectively, after glyoxal treatment (Fig. 5d).

**Fig. 6:**
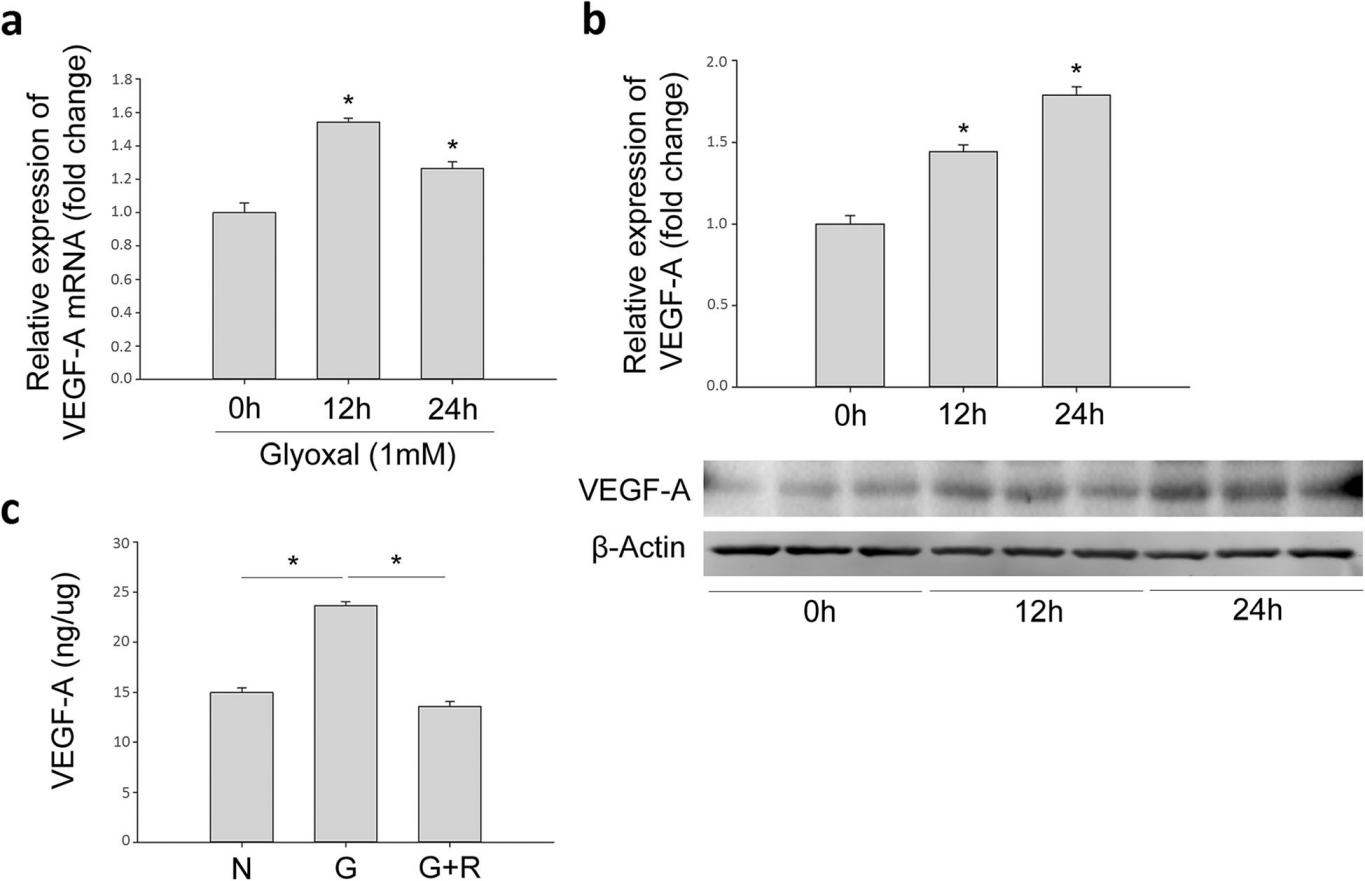
Expression of VEGF-A increased in rMC-1 cells when treated with glyoxal. The (**a**) mRNA and (**b**) protein level of VEGF-A in glyoxal-treated rMC-1 cells for 12 and 24 h. **c** The concentration of VEGF-A in the supernatant of glyoxal-treated rMC-1 cells with or without ranibizumab. Data are expressed as mean ± SE (n = 6 in [a], n = 3 in [b], n = 4 in [c], *P < 0.05). N: normal control; G: rMC-1 cells treated with 1 mM glyoxal; G + R: rMC-1 cells treated with glyoxal and ranibizumab

To study the effect of ranibizumab on rMC-1 cells, glyoxal-treated rMC-1 cells were treated with or without ranibizumab and the mRNA and protein levels of VEGF-A, Kir4.1, AQP4, Dp71 and GS were evaluated. Although cell viability was decreased in a time-dependent manner with glyoxal treatment (Fig. 5b), VEGF-A expression was increased at both 12 and 24 h (Fig. 6a and b). The mRNA level of VEGF-A was increased by 54.4 and 26.4% at 12 and 24 h, respectively, in the glyoxal-treated group (Fig. 6a). VEGF-A protein level was increased by 44.4 and 78.9%, respectively, at the same time points (Fig. 6b). VEGF-A level in the supernatant of cell culture decreased significantly after ranibizumab treatment (Fig. 6c).

**Fig. 7:**
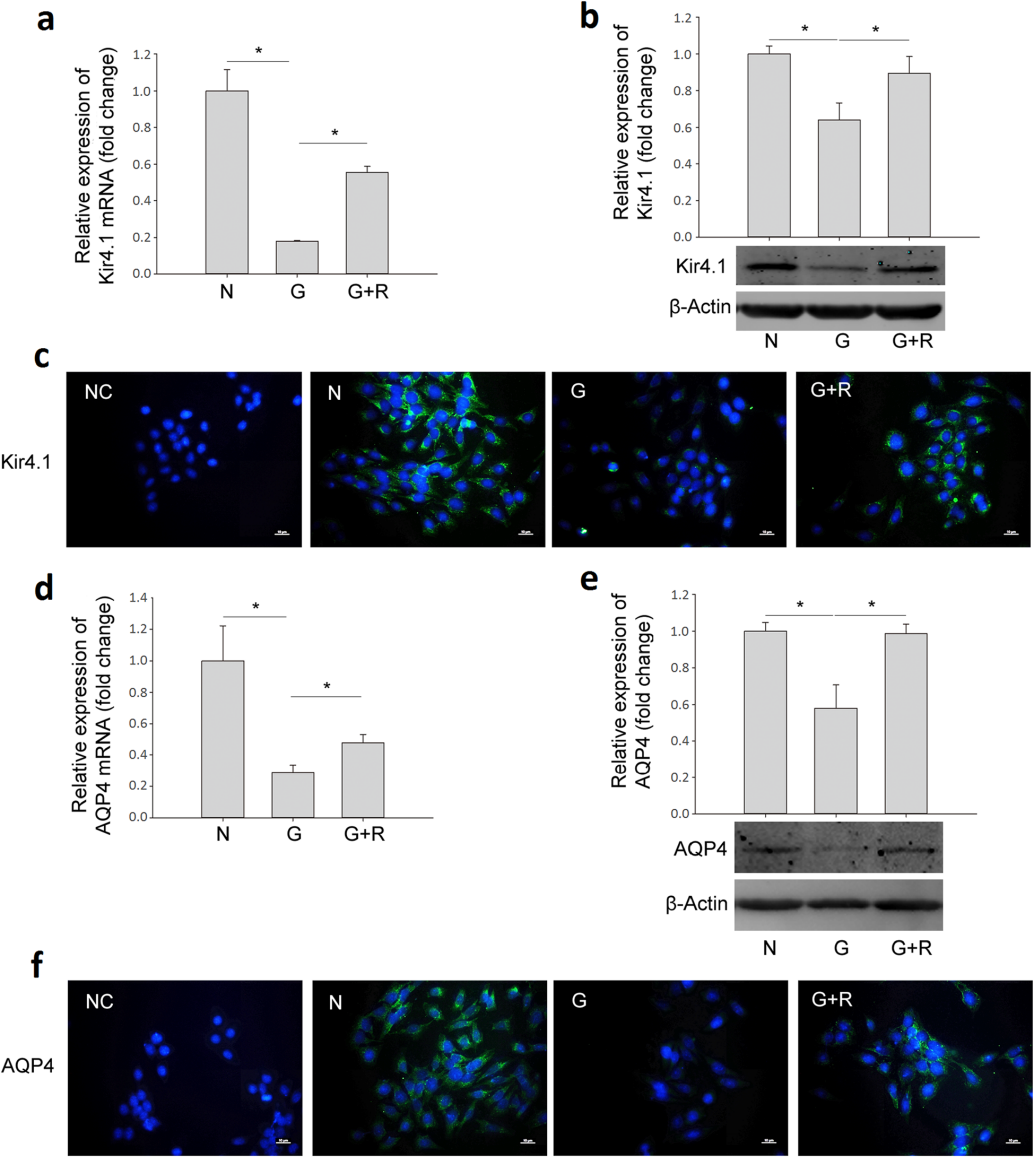
Changes in mRNA and protein levels of Kir4.1 and AQP4 in glyoxal-treated rMC-1 cells with or without ranibizumab. The (**a**) mRNA and (**b**) protein level of Kir4.1 in glyoxal-treated rMC-1 cells with or without ranibizumab. **c** Immunofluorescence of Kir4.1 in rMC-1 cells. The (**d**) mRNA and (**e**) protein level of AQP4 in glyoxal-treated rMC-1 cells with or without ranibizumab. **f** Immunofluorescence of AQP4 in rMC-1 cells. Data are expressed as mean ± SE (n = 8 in [a, d], n = 4 in [b, e], *P < 0.05). N: normal control; G: rMC-1 cells treated with 1 mM glyoxal; G + R: rMC-1 cells treated with glyoxal and ranibizumab. Scale bar: 10 μm

**Fig. 8:**
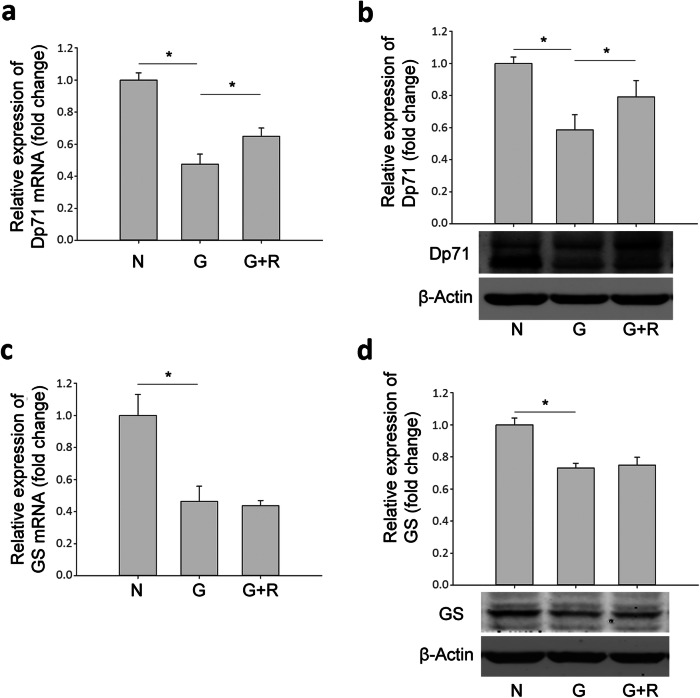
Changes in mRNA and protein levels of Dp71 and GS in glyoxal-treated rMC-1 cells treated with or without ranibizumab. The (**a**) mRNA and (**b**) protein level of Dp71 in glyoxal-treated rMC-1 cells with or without ranibizumab. The (**c**) mRNA and (**d**) protein level of GS in glyoxal-treated rMC-1 cells with or without ranibizumab. Data are expressed as mean ± SE (n = 8 in [a, c], n = 4 in [b, d], *P < 0.05). N: normal control; G: rMC-1 cells treated with 1 mM glyoxal; G + R: rMC-1 cells treated with glyoxal and ranibizumab

The mRNA levels of Kir4.1, AQP4, Dp71 and GS (Fig. 7 and Fig. 8) also decreased significantly in the glyoxal-treated group, i.e., decreased by 82.2% (Kir4.1), 71.1% (AQP4), 52.6% (Dp71) and 53.6% (GS), respectively; which were increased by 210.4% (Kir4.1), 65.0% (AQP4), 36.9% (Dp71) and decreased by 5.7% (GS), respectively, after ranibizumab treatment. The changes in protein expression followed a similar pattern. The protein levels of Kir4.1, AQP4, Dp71, and GS (Fig. 7 and Fig. 8) decreased by 36.0% (Kir4.1), 42.2% (AQP4), 41.4% (Dp71) and 26.9% (GS), respectively, in the glyoxal-treated group, which were increased by 39.5% (Kir4.1), 70.5% (AQP4), 34.9% (Dp71) and 2.4% (GS), respectively, after ranibizumab treatment. The changes in Kir4.1 (Fig. 7c) and AQP4 (Fig. 7f) were also confirmed with immunofluorescence.

### Exogenous VEGF-A decreased the expression of Kir4.1 in rMC-1 cells

**Fig. 9:**
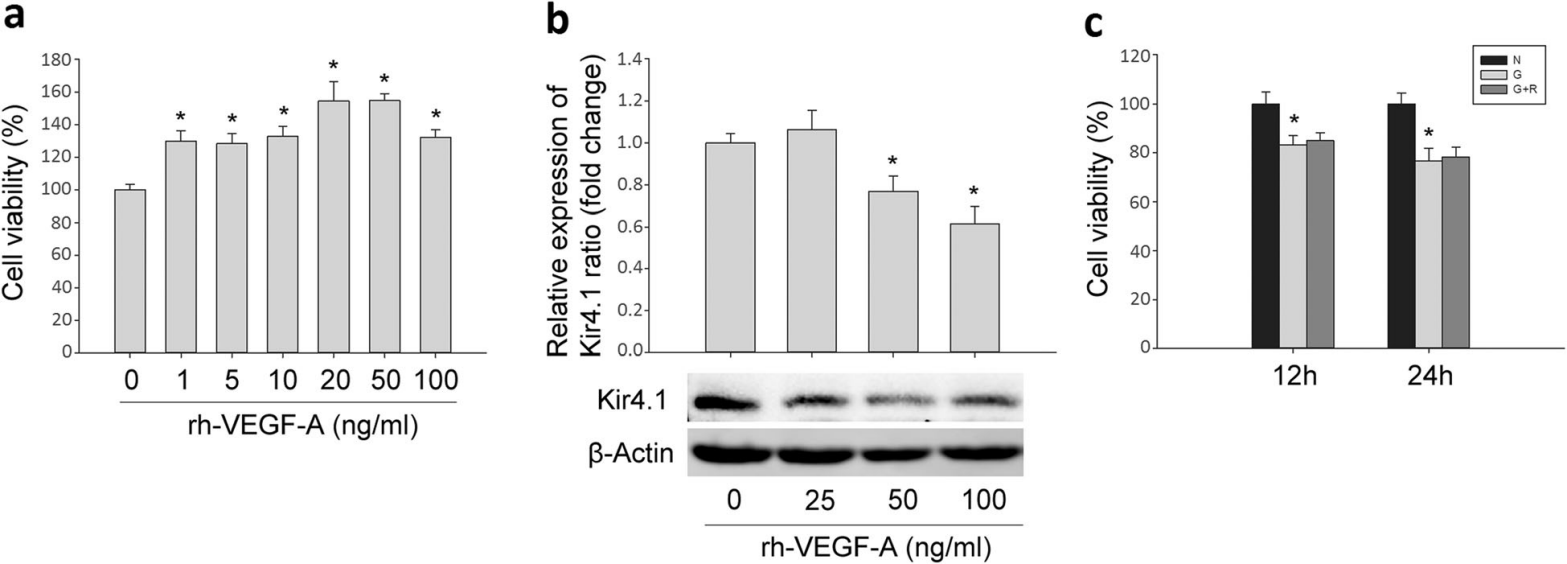
Exogenous VEGF-A decreased the expression of Kir4.1 in rMC-1 cells. **a** Cell viability of rMC-1 cells treated with rh-VEGF-A. **b** Protein expression of Kir4.1 in rMC-1 cells treated with rh-VEGF-A. **c** Cell viability of rMC-1 cells treated with glyoxal with or without ranibizumab. Data are expressed as mean ± SE (n = 12 in [a, c], n = 4 in [b], *P < 0.05). N: normal control; G: rMC-1 cells treated with 1 mM glyoxal; G + R: rMC-1 cells treated with glyoxal and ranibizumab

To study whether the increased VEGF-A in glyoxal-treated rMC-1 cells could decrease Kir4.1 expression, we treated rMC-1 cells with recombinant human VEGF-A (rh-VEGF-A). In Fig. 9a, cell viability was increased significantly with different doses of rh-VEGF-A treatment, e.g. cell viability increased by 29.8% (1 ng/mL), 32.9% (10 ng/mL) and 32.4% (100 ng/mL). Protein expression levels of Kir4.1 was decreased dose-dependently by rh-VEGF-A, i.e., decreased by 23.1% (50 ng/mL) and 38.6% (100 ng/mL), indicating that the down-regulation of Kir4.1 might be partially caused by increased VEGF-A in glyoxal-treated rMC-1 cells (Fig. 9b). Since ranibizumab has no effect on cell viability (Fig. 9c), the increased Kir4.1 by ranibizumab further confirmed the causal effect of VEGF-A on Kir4.1. We also detected the changes of AQP4 and Dp71 after rh-VEGF-A treatment but found no significant change for these two proteins (Data not shown).

### Ranibizumab decreased intracellular osmotic pressure by sodium efflux

**Fig. 10:**
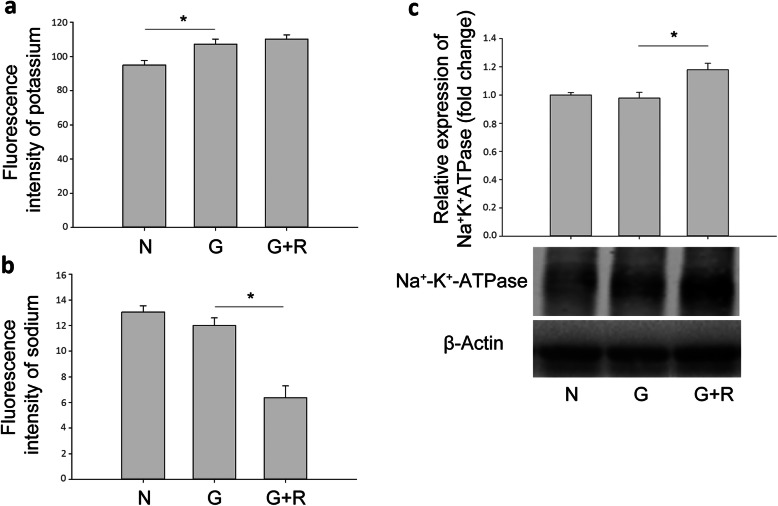
Ranibizumab decreased intracellular osmotic pressure by sodium efflux. **a** Intracellular potassium and (**b**) sodium levels were detected in glyoxal-treated rMC-1 cell with or without ranibizumab. **c** Protein expression of Na^+^-K^+^-ATPase in glyoxal-treated rMC-1 cells treated with or without ranibizumab. Data are expressed as mean ± SE (n = 10 in [a, b], n = 4 in [c], *P < 0.05). N: normal control; G: rMC-1 cells treated with 1 mM glyoxal; G + R: rMC-1 cells treated with glyoxal and ranibizumab

To test whether ranibizumab could prevent Müller cell from intracellular edema through decreasing osmotic pressure, we detected intracellular potassium and sodium levels using their corresponding indicators (PBFI and SBFI). After treatment with glyoxal (1 mM) for 24 h, the intracellular potassium level was increased significantly while the intracellular sodium level remained relatively unchanged compared with control (Fig. 10). However, when treated with ranibizumab, intracellular sodium level, but not potassium, decreased significantly. Thus, besides up-regulating Kir4.1, decreasing intracellular osmotic pressure might be another mechanism through which ranibizumab acts to prevent intracellular edema of Müller cells in DR. To further explore the possible reasons, we performed Western blot to detect protein expression of Na^+^-K^+^-ATPase in glyoxal-treated rMC-1 cells with or without ranibizumab treatment. From Fig. 10c, compared with normal control, the expression of Na^+^-K^+^-ATPase in glyoxal-treated group remained unchanged, while ranibizumab treatment increased its expression by 20.6%. The detailed mechanisms need further exploration.

## Discussion

DME is the main cause of blindness in patients with DR.^15^ Anti-VEGF therapy has been an effective treatment improving both microstructure and functions of the retina in DME patients. Further studying the underlying mechanisms of anti-VEGF therapy on DME and exploring other effective treatments are of great importance. In this study, we found that increased VEGF-A, and decreased Kir4.1, AQP4 and Dp71 in diabetic retinas contributed to the intracellular edema seen in Müller cells. Increased VEGF-A may be the initiator or causal factor for the down-regulation of Kir4.1, AQP4 and Dp71 because their altered expressions could be reversed when binding VEGF-A by ranibizumab. The ranibizumab's effect is independent of the gliotic state of Müller cells since ranibizumab showed no effect on both GS and GFAP expressions in Müller cells. Besides directly binding VEGF-A, ranibizumab could also decrease the intracellular sodium level to reduce the osmotic pressure, consequently preventing intracellular edema.

The pathogenesis of DME is complex. Breakdown of the inner BRB, and dysfunction of Müller cells and RPE were all involved in the pathogenesis of DME. Müller cell, like a pump, drains ions and water into the vitreous body and retinal blood vessels with the help of normal distribution and function of Kir4.1 and AQP4. It was reported that the distribution of Kir4.1 is altered in a 6-month diabetic rat retina, which is globally decreased especially in the OLM and around blood vessels.^22^ Another study found that Kir4.1 is absent in the perivascular areas and in the ILM in 3-month diabetic rats.^23^ However, most studies focused on the distributions of these channels using immunofluorescence but protein expression levels were rarely reported in DR. In this study, we found that the protein levels of Kir4.1 and AQP4 decreased significantly in the 12-week diabetic rat retina, and immunofluorescence of Kir4.1 was greatly decreased, especially at the endfeet of Müller cells, which is consistent with previous studies. These results indicate that the decreased expression levels as well as the redistribution of Kir4.1 and AQP4 might cause the dysfunction of Müller cells, which impaired water and ion transport out of the retina, and thus caused intracellular edema of Müller cells in DR. Up-regulation of Kir4.1 and AQP4 by dexamethasone in Müller cells protected the retina from edema in a surgically induced BRB breakdown model.^24^ These data support the important roles Kir4.1 and AQP4 play in the formation of intracellular edema in Müller cells. Other studies also reported that anti-VEGF treatment could up-regulate the expression levels of Kir4.1 and AQP4 on primary rat Müller cells.^25^ These results thus lay a foundation for the treatment of DME via the up-regulation of Kir4.1 and AQP4 in Müller cells.

Ranibizumab is a recombinant, humanized neutralizing antibody fragment, directly binding all isoforms of VEGF-A. It was verified as a powerful treatment to decrease macular edema in many clinical trials.^26^ Besides its binding to VEGF-A, the detailed mechanisms of clearing accumulated fluid were unclear. We hypothesized that, except for its effect on the BRB, anti-VEGF treatment might enhance the “pumping” ability of Müller cells to transport water and ion out of the retina via the retinal vasculature, maintaining the homeostasis of the retina. In this study, we found that ranibizumab, through the binding of VEGF-A, protected Müller cells from edema by up-regulating Kir4.1, AQP4 and Dp71.

Ion accumulation is considered the initial step for intracellular edema. The down-regulation of Kir4.1 could weaken efflux of potassium, causing potassium accumulation and increasing intracellular osmotic pressure. Water driven by osmotic pressure entered Müller cells through AQP4, leading to cell swelling. We found that the expression level of Kir4.1, not AQP4 or Dp71, decreased by rh-VEGF administration (Data not shown), indicating that the down-regulation of AQP4 and Dp71 in DR might be regulated by other factors, but not VEGF.

What was unexpected was that the intracellular potassium level was not decreased even though Kir4.1 was up-regulated by ranibizumab. On the other hand, intracellular sodium level decreased significantly after ranibizumab treatment. A possible explanation can be attributed to changes in Kir4.1 and Na^+^-K^+^-ATPase. Kir4.1 enables the efflux of K^+^ out of cells. Therefore, in glyoxal-treated rMC-1 cells, the increased K^+^ level (Fig. 10a) might be due to the decreased Kir4.1 expression (Fig. 7b) since the protein expression of Na^+^-K^+^-ATPase remained unchanged (Fig. 10c). After ranibizumab treatment, Na^+^ level was decreased (Fig. 10b), this might be due to the increased protein expression Na^+^-K^+^-ATPase by ranibizumab (Fig. 10c), which enhances the efflux of Na^+^. Meanwhile, the increased K^+^ influx caused by up-regulated Na^+^-K^+^-ATPase might be compromised by the up-regulated Kir4.1 (Fig. 7b), which increased K^+^ efflux after ranibizumab treatment, resulting in unchanged intracellular K^+^ level (Fig. 10a). The detailed mechanisms for ranibizumab in decreasing intracellular sodium level merits further study. Other potassium channels or ion channels might also be involved in ionic homeostasis of Müller cells, such as Kir2.1, a bidirectional potassium channel, which also plays an important role in regulating intracellular osmotic pressure.

Although anti-VEGF therapy is effective in treating DME, a weak correlation was reported between gain of visual acuity and the anatomical improvement.^27–30^ The loss of retinal neurons, especially cones, could result in the decreased visual acuity, which cannot be improved even after anatomical recovery. Further, the loss of retinal neurons might induce the gliotic reaction of Müller cells with overexpression of GFAP and downregulation of GS.^22, 31^ In our study, cell viability and expression of GFAP and GS were not influenced by ranibizumab in vivo and in vitro, indicating that ranibizumab has no effect on the gliotic reaction. Thus, it is of importance to develop combinatorial therapy to treat DME, e.g. to reduce retinal edema with anti-VEGF drugs while protecting retinal neurons with neurotrophic factors or regulating the gliotic state of Müller cells.

## Conclusion

Our data showed that the retinal protein expression levels of Kir4.1, AQP4 and Dp71 were decreased with diabetes progression and the distributions of these proteins were also changed, causing the dysfunction of Müller cells with intracellular edema in experimental DR. Ranibizumab protected Müller cells from intracellular edema via up-regulating the expressions of Kir4.1, AQP4 and Dp71 through binding VEGF-A. It could also increase the protein expression of Na^+^-K^+^-ATPase, thus decreasing the intracellular osmotic pressure of Müller cells. Hence, this study broadened our knowledge of the mechanisms of anti-VEGF therapy for DME and provided clues for treating DME through targeting Kir4.1 and AQP4.

## Highlights

1. The expressions of Kir4.1 and AQP4 were down-regulated in diabetic rat retina.
2. Ranibizumab increased the expression levels of Kir4.1 and AQP4 in diabetic rat retina and glyoxal-treated rMC-1 cells.
3. Ranibizumab binds VEGF-A to cause a decrease in intracellular edema by regulating sodium efflux.

## Data Availability

All data generated or analyzed during this study are included in this published article.
